# Designing and Evaluating Scalable Child Marriage Prevention Programs in Burkina Faso and Tanzania: A Quasi-Experiment and Costing Study

**DOI:** 10.9745/GHSP-D-19-00132

**Published:** 2020-03-30

**Authors:** Annabel Erulkar, Girmay Medhin, Eva Weissman, Gisele Kabore, Julien Ouedraogo

**Affiliations:** a Population Council, Addis Ababa, Ethiopia.; b Addis Ababa University, Addis Ababa, Ethiopia.; c Columbia University, New York, NY, USA.; d Pathfinder International, Ouagadougou, Burkina Faso.; e Christian Children's Fund of Canada, Ouagadougou, Burkina Faso.

## Abstract

**Background:**

A significant number of girls are married as children, which negatively impacts their health, education, and development. Given the sheer numbers of girls at risk of child marriage globally, the challenge to eliminate the practice is daunting. Programs to prevent child marriage are typically small-scale and overlook the costs and scalability of the intervention.

**Implementation:**

This study tested and costed different approaches to preventing child marriage in rural Burkina Faso and Tanzania. The approaches tested were community dialogue, provision of school supplies, provision of a livestock asset, a model including all components, and a control arm. A quasi-experimental design was employed with surveys undertaken at baseline and after 2 years of intervention. We examined the prevalence of child marriage and school attendance controlling for background characteristics and stratified by age group. Programmatic costs were collected prospectively.

**Results:**

Among those in the community dialogue arm in Burkina Faso, girls aged 15 to 17 years had two-thirds less risk (risk ratio [RR]=0.33; 95% confidence interval [CI]=0.19, 0.60) of being married and girls aged 12 to 14 years had a greater chance of being in school (RR=1.18; 95% CI=1.07,1.29) compared to the control site. In Tanzania, girls aged 12 to 14 years residing in the multicomponent arm had two-thirds less risk of being married (RR=0.33; 95% CI=0.11, 0.99), and girls 15 to 17 in the conditional asset location had half the risk (RR=0.52; 95% CI=0.30, 0.91). All the interventions tested in Tanzania were associated with increased risk of girls 12 to 14 years old being in school, and the educational promotion arm was also associated with a 30% increased risk of girls aged 15 to 17 years attending school (RR=1.3; 95% CI=1.01, 1.67). Costs per beneficiary ranged from US$9 to US$117.

**Conclusion:**

The study demonstrates that minimal, low-cost approaches can be effective in delaying child marriage and increasing school attendance. However, community dialogues need to be designed to ensure sufficient quality and intensity of messaging. Program managers should pay attention to the cost, quality, and coverage of interventions, especially considering that child marriage persists in the most hard-to-reach rural areas of many countries.

## Abstract (precis)

Minimal, low-cost approaches can be effective in delaying child marriage and increasing school attendance. Program managers should consider the cost, quality, and coverage of interventions, especially because child marriage persists in the most hard-to-reach, rural areas of many countries.

## INTRODUCTION

Child marriage has powerful negative implications for a young woman's health and well-being as it frequently marks the beginning of sexual activity and early childbearing, as well as an end to educational pursuits.^1^ Globally, child marriage is a widespread problem, especially in the developing world. Currently, 650 million women and girls were married before they turned 18 years old, with 12 million underage girls married each year.^2^^,^^3^ If efforts to prevent child marriage are not intensified, United Nations Children's Fund (UNICEF) estimates that 150 million more girls will be married by 2030.^4^ Girls affected by the practice are largely from the poorest families in the poorest and most remote rural places. In sub-Saharan Africa—where 38% of the child marriages are found—countries where significant proportions of girls marry before they are 18 years old are Niger (76%), Central African Republic (68%), Chad (67%), Burkina Faso (52%), Mali (52%) and South Sudan (52%).^3^

Existing evidence on interventions suggests that child marriage is not an intractable practice and that dedicated programs can have positive impacts on the practice. Several meta-analyses and evaluations of individual programs have examined what works in delaying child marriage. In 2012, Lee-Rife et al undertook a systematic review of evaluated child marriage interventions in developing countries. Among the 23 evaluated programs, only 4 focused on preventing child marriage as the primary objective and only 5 studies were undertaken in sub-Saharan Africa. Although evidence was limited, the review found that the most effective approaches in delaying child marriage were those that offered incentives and those that are directed at girls, providing them knowledge, skills, and opportunity to expand their social networks.^5^ More recent reviews explored both published and gray literature evaluations of child marriage prevention. These reviews found most of the successful programs included a conditional cash transfer or other provisions to allay the cost of schooling such as provision of school supplies, findings that essentially support those of the earlier review.^6^^,^^7^ Another review of rigorously evaluated projects found that empowerment approaches (approaches that give girls information, skills, and social support) were the most effective in reducing child marriage. In contrast to previous reviews, this review found economic approaches such as conditional cash/asset transfers to be of limited effectiveness.^8^

Given the sheer numbers of girls at risk of child marriage globally, the challenge to accelerate elimination of the practice is daunting. Pilot programs to prevent or reduce child marriage, whether evaluated or not, typically overlook the costs and scalability of the intervention design.^8^ Child marriage takes place on a massive scale, and responses must also be at-scale. However, most child marriage prevention programs remain at a small scale, led by nongovernmental organizations (NGOs) and reach fewer than 50,000 girls.^9^^–^^11^ The benefits of addressing child marriage are significant; the World Bank estimates that eradicating child marriage by 2030 will result in global benefits of over US$600 billion dollars.^12^ Child marriage largely occurs in the poorest countries with the greatest resource constraints. Therefore, is it incumbent upon program managers and researchers to not only ask what interventions work in preventing child marriage but also what package of interventions can reach the most girls at the least cost and still be effective in preventing the practice? For example, interventions such as the Zomba scheme in Malawi offered households a conditional cash transfer of between US$4 to US$10 per month and girls between US$1 to US$5 dollars per month.^13^ Although the scheme was found to be effective, it is unclear if such interventions can be scaled up in poor countries, given the cost of the intervention when offered to large numbers of families and girls.

### Berhane Hewan

In Ethiopia, *Berhane Hewan* (Amharic for “Light for Eve”) was one of the early child marriage prevention programs to include a rigorous, quasi-experimental evaluation. The program was a multicomponent package that included (1) community conversations to address social norms related to child marriage; (2) provision of school supplies to encourage school enrollment and thereby protect girls from marriage; (3) a conditional asset transfer, promising girls and their families a goat if they remain unmarried and in school; and (4) girls' groups led by adult women mentors that were mainly attended by married girls. After 2 years of intervention, girls aged 10 to 14 years in the Berhane Hewan site were one-tenth as likely to be married and 3 times more likely to be in school compared to girls residing in the control area.^14^

Given the success of Berhane Hewan in delaying marriage age, it was anticipated that government and NGO partners would scale up the approach to other locations in Ethiopia where child marriage was prevalent. However, in-country partners questioned the feasibility of scaling up the project given the complexity of its multicomponent design and wanted more information about the costs associated with scaling up the program over larger populations.^15^

As a result, a second generation of research was designed related to child marriage prevention. We sought to examine if streamlined, low-cost interventions could be effective in delaying marriage in sub-Saharan Africa, and at what cost? In this phase, we adapted the interventions used in the original Berhane Hewan project but implemented them separately in different geographical areas, with the intention to isolate the specific impact of streamlined interventions. During this research phase, costing data were collected for all interventions, and the program was expanded beyond Ethiopia to include the Cascades province of Burkina Faso and the Tabora region of Tanzania.

In this article, we present implementation, impact results, and costing data for child marriage prevention schemes in Burkina Faso and Tanzania only. Although we included research sites in the Amhara region of Ethiopia, where the Berhane Hewan project had been tested, we had to eliminate the control site because it became a resettlement area for people from other parts of the country. As a result, the population in this area was frequently unfamiliar with each other, making it significantly different from the other intervention sites. Marriage arrangements in Ethiopia are rooted in family ties and the desire to strengthen bonds between families who are essentially known to each other and/or neighbors. The control site included a resettled population, many of whom were strangers to each other or from different religions, inherent differences that could not be captured in the survey. As such, the marriage market in this area was compromised and rates of marriage were extremely low and uncharacteristic of the region.

## STUDY LOCATIONS

Burkina Faso is a francophone West African country where the prevalence of child marriage is the eighth highest in the world (52% married by age 18 years).^3^ Polygamous unions are common, and age differences between married girls and their husbands can be significant: on average 10.9 years among ever-married women aged 20 to 24 years.^16^ The Cascades Region is 1 of 13 administrative regions in Burkina Faso and borders Cote d'Ivoire and Mali. With a population of nearly 525,000, the region is largely agricultural and has cotton as a major economic activity. According to the 2010 Demographic and Health Survey (DHS) for Burkina Faso, 58% of girls aged 20 to 24 years in the Cascades region were married by their 18th birthday and 10% were married by age 15 years. Among women aged 20 to 24 years in the Cascades region, 64% have never been to school and only 15% have attained a secondary level education.^16^

The Tabora region is located in the central-western part of Tanzania and has a population of more than 2.2 million. The largely agricultural region has tobacco production as a major economic activity. Based on the 2010 Tanzania DHS, 63% of Tabora girls are married by age 18 years and 16% are married by age 15 years. Among girls aged 20 to 24 years, 42% have never been to school and only 9% have reached the secondary level.^17^

## PROGRAM DESCRIPTION

This study tested and costed streamlined interventions to delay the age at marriage in Burkina Faso and Tanzania. Local NGO partners implemented different child marriage prevention strategies derived from the Berhane Hewan program in Ethiopia in different geographical areas of the project regions. In each country, 4 approaches were implemented: (1) addressing community attitudes related to child marriage through community dialogue (2) providing unmarried girls aged 12 to 17 years with school materials, (3) offering a conditional asset transfer in the form of a goat, both conditioned on the 12-to-17-year-old girl remaining unmarried and in school for the duration of the pilot period (27–28 months), and (4) offering all components: community dialogue, school supplies and a conditional asset transfer. A fifth area served as a control where no intervention took place.

This study tested and costed streamlined interventions to delay the age at marriage in Burkina Faso and Tanzania.

The interventions were similar between regions but not identical. In each of the countries, modest modifications and adaptations were made by local implementing partners based on experiences and preferences as well as local circumstances, and so as not to impose approaches that might not be appropriate to the context (Table 1). Interventions were implemented for 28 months in Tanzania and 27 months in Burkina Faso.

**TABLE 1.** Overview of Child Marriage Prevention Program Interventions, by Model, Burkina Faso and Tanzania

	Burkina Faso	Tanzania
**Community dialogue**	—Facilitators recruited from communities and trained for five days. —Facilitators mobilize a cross-section of the community in groups of 30. —Groups attend 16 sessions. —Sessions include information on child marriage and girls' education and devising and implementing solutions.	—Community and religious leaders recruited and trained for two days. —Leaders deliver messages on child marriage and girls' education through routine community meetings such as religious services and community meetings.
**School supplies and clubs**	—Mentors go house-to-house to identify unmarried girls aged 12–17. —Parents and girls register to receive school supplies in public ceremony —Ceremonies used to educate public on importance of girls' education and eliminating child marriage. —Girls given school supplies once per year at the beginning of the school year. —Supplies were notebooks, pens, pencils, mathematical sets and US$3.50 subsidy of school fees.	—Mentors go house-to-house to identify unmarried girls aged 12–17. —Parents and girls register to receive school supplies in public ceremony —Ceremonies used to educate public on importance of girls' education and eliminating child marriage. —Girls given school supplies or school uniform once per year at the beginning of the school year. —Supplies were notebooks, pens, pencils, mathematical sets or a school uniform. —In-school girls' clubs formed including life skills and tutoring.
**Conditional asset transfer**	—Mentors go house-to-house to identify unmarried girls aged 12–17. —Parents and girls register to receive conditional asset (goat) in public ceremony —Goats awarded after two years if girls remained unmarried and in school. —Ceremonies used to educate public on importance of girls' education and eliminating child marriage.	—Mentors go house-to-house to identify unmarried girls aged 12–17. —Parents and girls register to receive conditional asset (goat) in public ceremony —Goats awarded after two years if girls remained unmarried and in school. —Ceremonies used to educate public on importance of girls' education and eliminating child marriage.
**Comprehensive model (all components)**	—All interventions above	—All interventions above
**Control**	No intervention	No intervention

### Addressing Community Attitudes Through Community Dialogue

Approaches to community dialogue differed in Burkina Faso and Tanzania with the approach in Burkina Faso being more systematic. In Burkina Faso, community facilitators were recruited from project communities and trained for 5 days using a facilitation manual. The manual contained information on facilitation techniques and on child marriage and girls' education. After training, facilitators mobilized community discussion groups composed of roughly 30 members containing a cross-section of the community: adult men, adult women, adolescent men, and adolescent women. Groups were taken through a 16-week curriculum that included both educational and action-oriented sessions. Education sessions provided information on the negative impact of early marriage and the value of girls' education. The action-oriented sessions provided a space for group members to devise home-grown solutions to child marriage and develop strategies to address it. Strategies could include house-to-house campaigns or systems of punishments or rewards for community members. Following the completion of the 4-month period, groups could have continued to meet on their own, if they desired. However, facilitators moved to additional areas to form new discussion groups. By the end of the program, 495 individual community members had taken part in the dialogues in Burkina Faso.

In the Tanzania community dialogue locations, community and religious leaders received 2 days of training on the benefits of delaying marriage and promoting girls' education. Leaders were trained on how to facilitate discussions and deliver messages and were asked to pass messages in their villages or places of worship. In contrast to the approach in Burkina Faso, the community dialogues in Tanzania did not include sustained and systematic contact with community groups but rather ad hoc contact with community members who attended routine community or religious meetings. Leaders in Tanzania did not record the number of community members who received their messages.

### Promoting Schooling Through Provision of School Supplies and School Clubs

Both in-school and out-of-school girls aged 12 to 17 years who were unmarried were eligible for the provision of school supplies, with out-of-school girls encouraged to return to school or join nonformal education. In school promotion locations, community-based mentors who were recruited and trained by the project identified eligible girls by going house-to-house. Once identified, project staff explained the scheme to girls and parents/guardians in the household and invited them to register for the scheme at a central location (mainly the local school) on a designated day. These explanations of the scheme were scripted so that information provided about the school promotion scheme was complete and uniform across households. On the day of registration and at the designated location, both the registering girl and her parent/guardian would sign the registration form in the presence of other community members and local leadership and take school materials with them with the agreement that the girl would remain unmarried and in-school for the duration of the pilot. Once it was confirmed that girls were attending school and remained unmarried, they were given her subsequent allocation of supplies in public ceremonies acknowledging their achievements by local leadership and school officials. These public ceremonies were also used as occasions to educate the community on the value of educating girls and keeping them unmarried.

Girls were given supplies once per year at the beginning of the school year, but the support differed by country. In Burkina Faso, registered girls received school supplies (notebooks, pens, pencils, and a mathematical set). In addition, they received a subsidy for school fees equivalent to US$3.50. In Tanzania, girls were given the choice of receiving a school uniform or school supplies. Tanzanian girls could attend Smart Girls' Clubs, which were after-school clubs that included life skills, reproductive health information, and tutoring. In all, 1,055 girls registered for the schooling program in Burkina Faso and 1,617 registered in Tanzania.

### Conditional Asset Transfer

Girls were recruited for the conditional asset transfer through community-based mentors going house-to-house to identify unmarried girls aged 12 to 17 years. Girls and their parents/guardians were sensitized on the scheme through a scripted narrative and asked to come to a central registration site on a specified day, if they wanted to enroll. In both countries, families and girls were awarded a goat at the end of the pilot period if the registered girls remained unmarried and in-school throughout the intervention period. In Tanzania, a pregnant goat was preferred because project partners perceived that such an asset would be further incentivizing to project communities. At the end of the pilot period, girls who remained unmarried and in school were awarded their livestock in a public ceremony with all the community members and local officials. As with the school material ceremonies, these occasions were used to educate the community on the value of girls' education. Seven hundred and thirty-two girls registered in Burkina Faso compared to 805 in Tanzania.

### Comprehensive, Multicomponent Model Site

In both countries, 1 location included all the project interventions: community dialogue, school promotion, and conditional asset transfer. All interventions we tested were designed to change social norms related to child marriage in different ways. The community dialogues directly addressed attitudes and beliefs related to child marriage and the status and value of girls. In addition, promotion of schooling and provision of conditional asset transfers were all implemented through public events under the auspices of local community leadership. These events raised the visibility and status of girls in the community and included messages from local leaders emphasizing the value delaying marriage and girls' education.

## METHODS

### Research Design

This was a quasi-experimental study design with cross-sectional baseline and end line surveys undertaken among girls aged 12 to 17 years. These surveys were used to measure population-level prevalence of child marriage and school attendance associated with the different interventions, using the control to measure changes that would have taken place without the interventions. Separate, cross-sectional baseline and end line surveys were undertaken before the interventions were established and after 27 to 28 months of intervention implementation.

The sample size was calculated to detect a 10-percentage point change in the prevalence of child marriage and adjusted for nonresponse and design effect. In each country, 2,500 girls aged 12 to 17 years were sampled, 500 per intervention area, at each round of survey. In each study commune (Burkina Faso) and ward (Tanzania), we randomly selected 17 enumeration areas from the existing national sampling frame. Each selected enumeration area underwent a household listing to establish a sampling frame. All members of the household were listed. Using the random number function in SPSS, we selected 30 girls aged 12 to 17 years per enumeration area. Only 1 respondent was interviewed per household. Where there was more than 1 eligible respondent per household, we used a Kish grid to select just 1 respondent.

Female interviewers were recruited from the project regions, ensuring that they were familiar with local languages and customs. At each survey round, interviewers received a 5-day training that included review of the questionnaire, review of ethical procedures, and practice interviews between interviewers and with girls recruited from outside the study areas.

Interviewers approached sampled respondents for a one-on-one interview. Similar to the DHS, interviewers made up to 3 visits to the household to locate and interview the sampled respondent. There was no replacement if the sampled respondent could not be located or refused to participate. Informed consent for the interviews was obtained from the parent or guardian of the sampled adolescent, and informed assent was obtained from the girl. If a girl was married, we considered her as an emancipated minor who could provide her own consent.

The questionnaire used in the study was a structured instrument covering areas such as demographic characteristics and living conditions, education, families and parent-child relationships, livelihoods, attitudes toward marriage and the experience of marriage, sexual experience, family planning, maternal and child health, and HIV and AIDS. A separate, abbreviated questionnaire was administered to younger respondents aged 12 to 14 years. This questionnaire omitted especially sensitive or potentially upsetting questions such as those related to violence or sexual coercion. The questionnaires were the same across countries at baseline and end line, except for country-specific modifications such as response codes for ethnic groups or religion. However, at end line, an additional section was added at the end to measure exposure to the interventions. The study received ethical clearance from the host institution's review board as well as local review boards in each of the study countries.

### Measures

The main outcome indicators measuring the impact of interventions was the percentage of girls who had ever been married and the percentage of girls in school in the current or previous year. Marriage was measured by asking the question, “Have you ever been married or lived with a person as married?” and a follow-up confirmatory question, “Have you ever been married but later divorced?” Girls who answered yes to either of these questions were coded as having been married and were asked a series of follow-up questions related to their husband and the timing and nature of their marriage.

To indicate whether girls had attended school in the last year, we asked respondents if they had ever been to school, if they were currently in school, and, if not, the age at which they left school. We used these 3 variables as well as the respondent's current age to construct a dichotomous variable reflecting school attendance in the previous year. We considered not only girls who were currently in school but also those who recently dropped out from school. This was because school supplies were only provided once per year at the beginning of the school year. Our field reports suggested that some girls in the 2 project countries dropped out of school in the second year of implementation after receiving their supplies because they knew no further supplies were forthcoming.

Multivariate models controlled for age, ethnicity, household assets as a reflection of socioeconomic status, and religion. Household socioeconomic status of respondents was measured based on ownership of a list of assets derived from DHS surveys. Each of these assets were given weights varying from 1 to 5, with the larger weight indicating greater value of the asset. Hence, car/truck was given a weight of 5; motorcycle/scooter was given a weight of 4; television, refrigerator, bicycle, and animal drawcart were given weights of 3; clock or watch, radio, telephone, mobile phone, and solar panel were given weights of 2; and table, chair, bed, and mattress were given a weight of 1. In each country, a weighted asset score was calculated after which respondents were scored as low-, middle-, or high-economic status. For religion, a dichotomous variable was created in both countries to reflect if the respondent was a Muslim or another religion.

Exposure and participation in the interventions were measured by asking respondents 2 separate questions. First, they were asked if they had heard of a project in their area offering community dialogues, school supplies, or a goat. In Tanzania, we also asked if girls had heard about a program offering girls' clubs in school. The second question asked if they had participated in the program including community dialogues, school supplies, or a goat. Those who had heard of and reported participation were coded as having participated in the project. Because the community dialogue arm included interventions that were targeted to both girls and the community at-large, respondents who had either heard of or participated in community dialogue were coded as having been exposed to the project. As the intervention was targeted to the community and not necessarily intended for the girl, it was assumed that those who had heard of the community dialogue had family and community members exposed to its messages. To measure exposure to the comprehensive model, we considered only residents of the study cell who had been exposed to all 3 project components as having been exposed to the comprehensive model. Follow-up questions were asked to assess levels of exposure to the interventions such as how many times they received school materials and how many community meetings they attended.

### Analysis

The analysis of the demographic characteristics was based on the unweighted sample. All other analyses were adjusted for the number of eligible females in the household to compensate for unequal probabilities of selection. Weighted numbers were reported. Exposure to the interventions is reported for each of the respective project sites at end line. In each of the countries, 1 study area was assigned to each model tested, due to logistical constraints. We undertook tests of equivalence using a confidence interval approach to understand the comparability of the study cells in each country.^18^^,^^19^ Through this approach, we assessed if intervention sites were within ±20% of the control site on various background measures. Sites with estimates that fell within ±20% of the control site estimate were considered to be equivalent in terms of that characteristic. We highlighted areas in which experimental sites were either equivalent or nonequivalent to the control site and controlled for all characteristics in the multivariate models. Additional analysis demonstrated that the conditional asset transfer site in Burkina Faso was significantly different from the control site, even after controlling for background factors. Thus, the conditional asset arm was eliminated from the analysis of results from Burkina Faso.

To estimate the impact of the interventions, we use Poisson regression to model risk ratios (RR) of having been married and having attended school in the last year, controlling for age, religion, ethnicity and low-, middle- or high-socioeconomic status. We stratified analysis by younger (aged 12 to 14 years) versus older (aged 15 to 17 years) adolescents. This stratification was done because the Berhane Hewan study demonstrated that interventions may operate differently among younger versus older adolescents, reflecting the divergent experiences and circumstances of older versus younger adolescents. At the same time, our sample was not powered to account for stratification. Separate models are presented for each intervention site, with reference to the control site, presenting RRs and 95% confidence intervals (CI) for residence in the intervention sites versus the control site, at end line. In addition, we did not assess the child marriage outcome for girls aged 12 to 14 years in Burkina Faso because there were too few cases of marriage among this age group at both baseline and end line.

### Management Information Systems

Management information systems in each country tracked information on girls' participation in each of the study arms. When registering, basic demographic information about each girl was entered into the database including age, school attendance, and highest year of education attained. Girls participating in the community sensitization arm were not registered as this approach targeted the community at-large. Management information systems data were entered in each country in an Epi-data file and converted to SPSS for analysis.

### Costing Study

At the beginning of the study, a Microsoft Excel spreadsheet was developed by a costing expert to enable systematic compilation of all project costs. Cost categories included staff time, office expenditures, training and meeting costs, travel expenditure, and purchase of commodities. Costs associated with evaluation were not included. Cost data were updated in the spreadsheet on a monthly basis by project staff and our in-country partners. On a yearly basis, costing data were validated by the costing expert.

At the end of the intervention, total costs were calculated by country and model. Using data from the management information system on the number of girls served per model, we calculated the cost per girl served, per year. Because the community dialogue did not include only girls, we estimated the population reached using population estimates and the percentage of girls at end line hearing about or participating in community dialogue.

## RESULTS

### Sample Characteristics

Table 2 shows the demographic characteristics of girls interviewed at baseline in the 2 countries, by study cell. Only 47% of Burkinabe girls and 57% of Tanzanian girls were in school at the time of baseline. Religious composition varied considerably between countries. The study population in Burkina Faso was predominantly Muslim (85%). There was a greater religious mix in Tanzania with about half (55%) being Muslim, 37% Christian and 8% another religion. At baseline, 18% of Burkinabe girls had ever been married compared to 8% of Tanzanian girls. Characteristics of girls varied between study cells in many cases. Where characteristics were not comparable, these variables were adjusted for in regression models.

**TABLE 2.** Demographic Characteristics of Girls Aged 12 to 17 Years Surveyed at Baseline, by Country and Model^a^

Burkina Faso	Total, % (N=2421)	Control, % (n=504)	Community dialogue, % (n=321)	Education promotion,% (n=509)	Asset transfer, % (n=488)	Comprehensive model, % (n=599)
**School Status**						
In school	47.2	50.1	59.1^b^	50.8	33.0^b^	47.1
Out of school	52.8	49.9	40.9^b^	49.2	67.0^b^	59.9
**Religion**						
Christian	4.2	4.8	6.5^b^	3.7	3.3	3.5
Muslim	84.6	91.2	61.7^b^	92.7	87.5	82.1
Other^1^	11.2	4.0	31.8^b^	3.6	9.2	14.4
Ethnicity (% Senoufo)	67.9	62.6	65.4	36.1^b^	89.1^b^	83.1^b^
**Marital status**						
Never married	82.2	84.1	88.2^b^	80.7^b^	66.9^b^	90.9^b^
Ever married	17.8	15.9	11.8^b^	19.3^b^	33.1^b^	9.1^b^
**Asset based SES**						
Low	36.1	51.8	28.7^b^	43.9^b^	12.5^b^	39.5^b^
Middle	36.9	29.4	38.9^b^	36.2^b^	36.1^b^	43.5^b^
High	27.0	18.8	32.4^b^	19.9^b^	51.4^b^	17.0^b^
**Tanzania**	**Total (N=2133)**	**Control (n=414)**	**Community sensitization^b^ (n=398)**	**Education promotion^b^ (n=483)**	**Asset transfer^b^ (n=408)**	**Comprehensive model^b^ (n=430)**
School status						
In school	56.7	47.8	63.0	53.1	59.3	60.8
Out of school	43.3	52.2	37.0	46.9	40.7	39.2
Religion						
Christian	36.7	58.4	30.1	30.2	39.6	26.5
Muslim	55.3	17.9	64.1	64.6	57.0	71.2
Other^c^	8.0	23.7	5.8	5.2	3.4	2.3
Ethnicity (% Mnamezi)	54.3	31.9	59.5	59.0	59.9	60.7
Marital status						
Never married	91.9	88.4	91.7	93.4	91.8	93.7
Ever married	8.1	11.6	8.3	6.6	8.2	6.3
Asset-based socioeconomic status						
Low	44.1	54.6	39.7	42.4	39.0	44.9
Middle	28.8	23.2	30.7	31.9	27.2	30.5
High	27.1	22.2	29.6	25.7	33.8	24.6

a Unweighted data.

b Measures not equivalent using confidence interval tests of equivalence.

c No religion or other/traditional religion.

Table 3 shows the percentage of girls at end line who were exposed or took part in the interventions in each study arm, by country. Overall, interventions achieved better coverage in Tanzania than in Burkina Faso. Exposure to the interventions varied considerably between countries. In Burkina Faso, the 2 interventions that achieved the most coverage were conditional asset transfer (54% of girls enrolled), followed by community dialogue/sensitization (48% of girls either participating or hearing of meetings). Schooling promotion received the most coverage in Tanzania (91%) followed by community sensitization (89%). The comprehensive model with multiple components achieved among the lowest coverage (20% in Burkina Faso and 52% in Tanzania).

**TABLE 3.** Girls Aged 12 to 17 Years Exposed to the Child Marriage Prevention Interventions, at End Line, by Study Arm and Country^a^

	Burkina Faso, % (N=2,655)	Tanzania, % (N=2,324)
Community dialogue/sensitization	47.9	89.4
School promotion: school materials	30.6	90.7
School promotion: ‘Smart Girl Clubs' (Tanzania only)	-	50.7
Conditional asset transfer	53.9	52.0
Comprehensive model	20.2	52.3

a Weighted data and weighted numbers presented.

### Child Marriage

We modelled the RRs at end line associated with being married in each of the study communities in Burkina Faso and Tanzania, with reference to the control site. In Burkina Faso, we did not examine the impact on marriage age among younger girls aged 12 to 14 years, as too few girls were married at both baseline (12 married girls) and end line (5 married girls). As previously mentioned, the conditional asset transfer arm was removed from analysis because of incomparability of that site, even after controlling for background factors. In Burkina Faso, girls aged 15 to 17 years who were enrolled in the community dialogue arm were one-third as likely to be married as those in the control site (RR=0.33; 95% CI=0.19, 0.60) (Table 4).

**TABLE 4.** Risk Association of Intervention for Burkinabe and Tanzanian Girls Having Ever Been Married at End Line, With Reference to the Control Group, by Study Arm and Age Group^a^

	Aged 12 to 14 Years, RR^b^ (95% CI)	Aged 15 to 17 Years, RR^b^ (95% CI)
**Burkina Faso**		
Community dialogue	—	0.33 (0.19, 0.60)^e^
Education promotion: school materials and fees	—	0.99 (0.66, 1.49)
Comprehensive model	—	0.77 (0.52, 1.15)
**Tanzania**		
Community sensitization	1.13 (0.48, 2.68)	0.74 (0.43, 1.27)
Education promotion: school materials and girls' clubs	0.70 (0.30, 1.65)	0.99 (0.64, 1.53)
Conditional asset transfer	0.41 (0.15, 1.15)	0.52 (0.30, 0.91)^c^
Comprehensive model	0.33 (0.11, 0.99)^c^	0.59 (0.34, 1.01)

Abbreviations: CI, confidence interval; RR, risk ratio.

a Weighted data.

b Adjusted for age, religion, ethnicity, and socioeconomic status.

c *P* < .05.

e *P* < .001.

In Tanzania, girls 12 to 14 residing in the comprehensive site had two-thirds less risk of being married compared to girls in the control site (RR=0.33; 95% CI=0.11, 0.99). Tanzanian girls 15 to 17 residing in the conditional asset transfer site had roughly half the risk of being married compared to girls in the control site (RR=0.52; 95% CI=0.30, 0.91).

We re-analyzed data to model RRs among Burkinabe and Tanzanian girls who reported that they had been exposed to the intervention at end line, with reference to the control site. Although selectivity issues undoubtedly impact upon results, the results of these analyses are consistent with the findings presented above (analysis not shown). In Burkina Faso, girls exposed or aware of the community dialogue had three-quarters less risk of being married (RR=0.27) compared to girls in the control site. Tanzanian girls aged 15 to 17 years residing in the conditional asset transfer arm and the comprehensive model had 90% less risk of being married compared to those in the control. This analysis demonstrates similar results among the exposed compared to the intention-to-treat analysis design, giving us confidence in the findings from our earlier analysis.

### School Attendance in the Current or Last Year

We examined differences in the risk of girls attending school in the current and previous years. We included attendance during the current year and previous year because school material was provided only once per year. We received information from staff in the field that some participants left school after receiving the second tranche of school supplies, knowing that additional supplies would not be provided in the next year. Table 5 shows the risk ratios and 95% CI for Burkinabe and Tanzanian girls attending school during the current and previous year, with reference to girls in the control site.

**TABLE 5.** Risk Association for Burkinabe and Tanzanian Girls Being in School in the Current and Previous Years at End Line, by Age Group^a^

	Age 12 to 14, RR^b^ (95% CI)	Age 15 to 17, RR (95% CI)
Burkina Faso		
Community dialogue	1.18 (1.07, 1.29)^c^	1.06 (0.94, 1.19)
Education promotion: school materials and fees	0.99 (0.89, 1.09)	0.92 (0.80, 1.05)
Comprehensive model	1.07 (0.96, 1.18)	1.03 (0.91, 1.15)
Tanzania		
Community sensitization	1.17 (1.04, 1.32)^c^	0.92 (0.68, 1.23)
Education promotion: school materials and girls' clubs	1.21 (1.08, 1.35)^c^	1.30 (1.01, 1.67)^d^
Conditional asset transfer	1.34 (1.20, 1.48)^e^	1.29 (1.00, 1.68)
Comprehensive model	1.28 (1.15, 1.43)^e^	1.00 (0.76, 1.32)

a Weighted data.

b Adjusted for age, religion, ethnicity, and socioeconomic status.

c *P* < .01.

d *P* < .05.

e *P* < .001.

Burkinabe girls aged 12 to 14 years residing in the community dialogue site had a greater chance of being in school (RR=1.2; 95% CI=1.07, 1.29) compared to their counterparts in the control. This finding is consistent with the fact that girls in this site were also less at risk for child marriage, which supports the effectiveness of this intervention in Burkina Faso. In Tanzania, all the interventions tested were associated with increased risk of girls aged 12 to 14 years being in school. The educational promotion arm was also associated with a 30% increased risk of being in-school among girls aged 15 to 17 years (RR=1.3; 95% CI=1.01, 1.67).

### Cost Analysis

Table 6 shows the cost per participating girl per year for the different intervention models. It should be noted that this is not cost-effectiveness but rather the cost to the program to implement the activities and support girls through various approaches. In both countries, community dialogue and school promotion cost roughly the same: from US$9 to US$12 per community member served for community dialogue and from US$13 to US$18 per girl served with school supplies. That the community sensitization was much less expensive in Tanzania compared to Burkina Faso probably reflects the less intensive and ad hoc nature of the intervention. In Tanzania, community leaders were trained to deliver messages but were not followed-up nor asked to report on the number of type of messages passed. In contrast, community dialogue in Burkina Faso involved intensive and systematic activities using paid facilitators to take community members through 4 months of structured discussions. The conditional asset transfer and the comprehensive models were the most costly to implement. In particular, provision of livestock and the comprehensive model was expensive in Tanzania, over US$100 per girl served. In Tanzania, the purchase, transport, and storage of goats added to the cost of implementation. In addition, program managers purchased more expensive pregnant goats, rather than the less expensive younger goats procured in Burkina Faso.

**TABLE 6.** Cost per Girl/Person Served per Year, by Model and Country

	Burkina Faso (US$)	Tanzania (US$)
Community sensitization/dialogue	12	9
School supplies and fees	13	–
Girls' clubs	–	18
Conditional asset transfer	33	107
Comprehensive model	60	117

## DISCUSSION

### Lessons Learned

Child marriage is considerably more common in rural areas compared to urban areas. Sites for this study were all rural and difficult to access. As such, the cost to deliver interventions in these rural areas was undoubtedly higher than similar efforts in urban areas. The coverage or reach of interventions varied. A much lower proportion of girls was exposed to the comprehensive model in both countries, likely reflecting the increased difficulty in implementing complex, multicomponent programs, exacerbated by the fact that interventions are being implemented in remote rural areas, where households are dispersed, isolated, or inaccessible. Although it had been expected that the comprehensive model would be more effective than simpler or single-component models in bringing about improved outcomes, the difficulty in implementing multicomponent programs in remote areas may have undermined the effectiveness of the intensive program model. Therefore, program implementers should closely monitor the coverage and quality of child marriage interventions. Specifically, they should weigh the trade-offs between more comprehensive program design that may be difficult to implement at scale in remote areas against simpler models that can more easily achieve coverage and scale and maintain quality.

Child marriage is commonly perceived as an intractable traditional practice. Many practitioners assume that complex and cost-intensive interventions are needed to achieve a tipping point. Our study results suggest that streamlined minimal approaches can be effective in delaying child marriage and encouraging school attendance. Community dialogue was effective in Burkina Faso, both in delaying child marriage and in increasing school attendance; however, the approach did not bring about measurable change in Tanzania. Community dialogue in Burkina Faso was systematically implemented using dedicated, paid facilitators who implemented a set curriculum among representatives of the community recruited for the dialogues. The approach in Tanzania was less rigorous and used existing community leaders to pass messages during routine community meetings. The ad hoc nature of the approach in Tanzania probably weakened the quality and intensity of messaging, as well as, possibly, cultural differences or differential receptivity to messages. This suggests that efforts to promote social norm change need to be implemented in a rigorous and systematic manner and not simply delegated to community members or religious leaders without rigorous training and follow-up.

Our study results suggest that streamlined minimal approaches can be effective in delaying child marriage and encouraging school attendance.

Finally, this is one of the few studies on child marriage prevention to include rigorous costing data. The study demonstrated that streamlined interventions can be effective to both delay marriage and contain costs, thus maximizing the likelihood for scale-up to large numbers of at-risk girls. Community dialogues in Burkina Faso cost only US$12 per person reached per year; schooling promotion in Tanzania cost only US$18 per girl supported per year. However, the cost of providing goats as a conditional asset transfer ranged from US$33 per girl served in Burkina Faso to US$107 per girl in Tanzania. The higher cost of purchasing pregnant goats in Tanzania compared with purchasing less expensive younger goats in Burkina Faso coupled with the delivery cost to remote areas of Tabora region drove up the cost of the conditional asset transfer in Tanzania. The high unit cost in Tanzania underscores the fact that decisions about programs and purchases should take cost into careful consideration to avoid excessive costs that undermine the chances of scalability. In addition, programmers should carefully consider the choice of economic incentives. A more recent program in Ethiopia eliminated livestock and introduced solar-powered lanterns as incentives to delay child marriage.^20^ Not only were the lanterns less expensive than livestock and easier to deliver, but they were used by recipients both to light homes and study in the evening and to generate income by charging cell phones for a small fee.

This is one of the few studies on child marriage prevention to include rigorous costing data.

Child marriage affects a large number of developing country girls, mainly in the poorest, most remote, and hard-to-reach locations. Programs designed to reach the greatest number of girls possible will require a greater number of impact evaluations coupled with costing studies. This study demonstrated that cost-contained interventions are feasible and can be effective in preventing child marriage. However, interventions need to take into account the quality and coverage of interventions, especially considering the difficulty of implementation in remote areas, where child marriage tends to persist.

### Limitations

The study had a number of limitations. Due to logistical and budgetary constraints, only 1 location was used to test and compare each model implemented. Including multiple sites per model tested would have likely increased comparability between sites and our confidence in the study results. Unfortunately, the control site in Ethiopia was found not to be comparable to the experimental sites, which weakened our ability to detect changes associated with interventions. In addition, the conditional asset transfer site in Burkina Faso was not comparable to the control site, even after controlling for background factors. As such, we could not assess the effectiveness of this approach. Lastly, based on past experience, it was necessary to stratify the analysis by age group. At the same time, the sample was not necessarily powered to accommodate this subgroup analysis.

## CONCLUSION

This study demonstrates that minimal, low-cost approaches can be effective in delaying child marriage and increasing school attendance. However, community dialogues need to be designed to ensure sufficient quality and intensity of messaging. Program managers should consider the cost, quality, and coverage of interventions, especially because child marriage persists in the most hard-to-reach, rural areas of many countries.

## Notes

**En français**

**Conception et évaluation des programmes de prévention des mariages des enfants prêts pour une mise à échelle au Burkina Faso et en Tanzanie: Une étude quasi-expérimentale et de calculs des coûts**

Des approches minimales et peu coûteuses peuvent être efficaces pour retarder le mariage des enfants et augmenter la fréquentation scolaire. Les gestionnaires de programmes doivent tenir compte du coût, de la qualité et de la couverture des interventions, notamment parce que le mariage des enfants persiste dans les zones rurales les plus difficiles d'accès de nombreux pays.

**RÉSUMÉ**

**Contexte**: Un grand nombre de filles sont mariées lorsqu'elles sont enfants, ce qui a un impact négatif sur leur santé, leur éducation et leur développement. Étant donné le nombre de filles qui risquent d'être mariées dans le monde, le défi pour éliminer cette pratique est immense. Les programmes visant à prévenir le mariage des enfants sont généralement de petite envergure et négligent les coûts et l'extensibilité de l'intervention.

**Mise en œuvre**: Cette étude a testé et chiffré différentes approches de prévention du mariage des enfants dans les zones rurales du Burkina Faso et de la Tanzanie. Les approches testées étaient le dialogue communautaire, l'allocation de fournitures scolaires, la fourniture de matériel d'élevage, un modèle incluant toutes les composantes et un bras de contrôle. Un modèle quasi-expérimental a été utilisé avec des enquêtes entreprises au départ et après deux ans d'intervention. Nous avons examiné l'évolution de la prévalence des mariages d'enfants et de la fréquentation scolaire, en contrôlant les caractéristiques du contexte et en les stratifiant par groupe d'âge. Les coûts du programme ont été recueillis de manière prospective.

**Résultats**: Parmi les participants au dialogue communautaire au Burkina Faso, les filles âgées de 15 à 17 ans avaient deux tiers de risque en moins (rapport de risque [RR]=0,33; intervalle de confiance [IC] de 95%=0,19, 0,60) d'être mariées et les filles âgées de 12 à 14 ans avaient une plus grande chance d'être scolarisées (RR=1,18; IC de 95%=1,07, 1,29) par rapport au site témoin. En Tanzanie, les filles âgées de 12 à 14 ans résidant dans le site à composantes multiples avaient deux tiers de risque en moins d'être mariées (RR=0,33; 95% IC=0,11, 0,99), et les filles de 15 à 17 ans dans le site à actifs conditionnels avaient la moitié du risque (RR=0,52; 95% IC=0,30, 0,91). Toutes les interventions testées en Tanzanie étaient associées à un risque accru de scolarisation des filles de 12 à 14 ans, et le volet promotion de l'éducation était également associé à un risque accru de 30% de scolarisation des filles de 15 à 17 ans (RR=1,3; 95% IC=1,01, 1,67). Les coûts par bénéficiaire allaient de 9 à 117 dollars américains.

**Conclusion**: L'étude démontre que des approches minimales et peu coûteuses peuvent être efficaces pour retarder le mariage des enfants et augmenter la fréquentation scolaire. Toutefois, les dialogues communautaires doivent être conçus de manière à garantir une qualité et une intensité suffisantes des messages. Les responsables de programmes doivent prêter attention au coût, à la qualité et à la couverture des interventions, surtout si l'on considère que le mariage des enfants persiste dans les zones rurales les plus difficiles à atteindre de nombreux pays.

**Peer Reviewed**
